# Optimizing Manufacturing Protocols of Chimeric Antigen Receptor T Cells for Improved Anticancer Immunotherapy

**DOI:** 10.3390/ijms20246223

**Published:** 2019-12-10

**Authors:** Sophia Stock, Michael Schmitt, Leopold Sellner

**Affiliations:** 1 Department of Internal Medicine V, Heidelberg University Hospital, 69120 Heidelberg, Germany; sophia.stock@med.uni-heidelberg.de (S.S.); michael.schmitt@med.uni-heidelberg.de (M.S.); 2 National Center for Tumor Diseases (NCT), German Cancer Consortium (DKTK), 69120 Heidelberg, Germany; 3 Oncology Business Unit—Medical Affairs, Takeda Pharma Vertrieb GmbH & Co. KG, 10117 Berlin, Germany

**Keywords:** chimeric antigen receptor, CAR, CART, adoptive cell therapy, immunotherapy, T lymphocyte, CART cell production, T cell activation, cytokines

## Abstract

Chimeric antigen receptor (CAR) T cell therapy can achieve outstanding response rates in heavily pretreated patients with hematological malignancies. However, relapses occur and they limit the efficacy of this promising treatment approach. The cellular composition and immunophenotype of the administered CART cells play a crucial role for therapeutic success. Less differentiated CART cells are associated with improved expansion, long-term in vivo persistence, and prolonged anti-tumor control. Furthermore, the ratio between CD4+ and CD8+ T cells has an effect on the anti-tumor activity of CART cells. The composition of the final cell product is not only influenced by the CART cell construct, but also by the culturing conditions during ex vivo T cell expansion. This includes different T cell activation strategies, cytokine supplementation, and specific pathway inhibition for the differentiation blockade. The optimal production process is not yet defined. In this review, we will discuss the use of different CART cell production strategies and the molecular background for the generation of improved CART cells in detail.

## 1. Introduction

Modern cancer therapies are increasingly relying on immunotherapeutic approaches. In particular, immune checkpoint inhibitors and adoptive cell therapy (ACT), including tumor-infiltrating lymphocytes (TILs), T cell receptor (TCR)-modified T cells, and chimeric antigen receptor (CAR) T cells represent milestones in innovative strategies for cancer treatment. ACT showed limitations, as the therapy with TILs only achieved encouraging results in selected highly immunogenic cancer entities, such as malignant melanoma [1]. Human leukocyte antigen (HLA)-restricted antigen recognition limits the application of TCR-modified T cells. The downregulation of HLA expression can lead to tumor escape [2]. CART cells combine the dynamic of T cells with the antigen-specificity of an antibody. They can bind the tumor antigen without antigen processing and independent of HLA-mediated antigen presentation. CD19-specific CART cell therapy showed very promising results in B cell malignancies, including acute lymphoblastic leukemia (ALL), chronic lymphocytic leukemia (CLL), and Non-Hodgkin lymphoma (NHL) [3]. Recently, the U.S. Food and Drug Administration (FDA) and the European Medicines Agency (EMA) approved *Kymriah^®^* (*Tisagenlecleucel*) for the treatment of patients with relapsed/refractory (r/r) B cell precursor ALL [4] or diffuse large B cell lymphoma (DLBCL) [5] and *Yescarta^®^* (*Axicabtagene Ciloleucel*) for the treatment of patients with r/r DLBCL and primary mediastinal B cell lymphoma (PMBCL) [6]. Additional tumor antigen targets are currently under development, such as B cell maturation antigen (BCMA), for the treatment of multiple myeloma [7]. In solid tumors, CART cells still have to overcome limitations in their therapeutic use [8].

Despite encouraging response rates, relapses occur and limit the efficacy of this promising treatment approach. Therefore, it is critical to understand the current limitations of CART cell therapy in order to utilize the full potential of this modern anticancer therapy [9,10]. The in vivo efficacy of CART cells is linked to their proliferative capacity and long-term persistence to sustain sufficient anti-tumor activity [11]. The in vivo expansion and persistence of CART cells is limited in certain patients and it prohibits long-term anti-tumor control. One approach for improving the activity of CART cells is the further development of CAR constructs and gene transfer systems. The reduced fitness and dysfunction of T cells in the applied final cell product of certain patients might be another reason for the impaired in vivo activity. Therefore, improving the mitochondrial fitness and biogenesis may enhance the therapeutic efficacy of CART cell therapy and other ACTs [12]. Another approach for improved therapeutic CART cell efficacy is the selection or modification of CART cell subpopulations and subsets. The cellular composition of the final cell product has a major impact on the proliferative capacity of CART cells and it is directly linked to in vivo efficacy [13,14,15]. Optimal T cell activation and cultivation strategies for CART cell generation are crucial in producing efficient CART cells with the preferred T cell immunophenotype and subsets. However, the manufacturing processes of CART cells are not yet standardized. In this review, different strategies for the generation of highly potent CART cells will be discussed.

## 2. The Role of Different T Cell Subtypes and Subpopulations for Efficient CART Cell Therapy

The number of transfused CART cells was assumed to majorly determine the therapeutic success in an early stage of the CART cell therapy. However, above a certain threshold, the absolute number of transfused CART cells does not directly correlate with in vivo expansion and therapeutic success [3]. Consequently, other factors than the absolute number of transfused CART cells might be more important for CART cell efficacy. For example, the cellular composition and phenotype of the adoptively transferred T cells, including T cell subtypes and subpopulations, was identified as one of the most critical success factors for efficient immunotherapy [16,17].

Although cytotoxic CD8+ CART cells, in particular, mediate direct tumor cell eradication, CD4+ T helper cells (Th cells) were identified as a highly potent and clinically important T cell subset [18]. It was demonstrated that CD4+ CART cells possess cytotoxic capacities that are comparable to cytotoxic CD8+ CART cells [19]. In addition, a balanced ratio of CD4+ Th cells and CD8+ cytotoxic T cells can positively influence the product regarding tumor eradication [13]. It was reported that the treatment of B-ALL patients with a 1:1 ratio of CD4+ and CD8+ (CD4:CD8 ratio) CART cells could achieve high remission rates [15]. For example, *Lisocabtagene maraleucel* (*liso-cel; JCAR017*) represents an anti-CD19 CART cell product administered in a defined composition with a specific ratio of CD4+ Th CART cells and cytotoxic CD8+ CART cells [20]. The subsets must be isolated at the beginning of the production and separately modified in order to gain a defined CD4:CD8 ratio, leading to a more complex manufacturing process.

Moreover, the different Th cell subpopulations play an important role. The balance between T_Eff_ cells and regulatory T (T_reg_) cells can influence the success of adoptive immunotherapy [21]. The infiltration of CD4+ T_reg_ cells into solid tumors can decrease the anti-tumor activity of CD28-CD3ζ signaling CART cells [22]. The deletion of the Lck binding moiety in the CD28 CAR endodomain of a CD28-CD3ζ signaling CAR can enhance the anti-tumor efficacy in the presence of T_reg_ cells [22]. It was reported that CART cells with the inducible T cell costimulator (ICOS) intracellular signaling domain can stabilize the Th17 cell function and enhance the in vivo persistence of CART cells in mice bearing human tumor xenografts [23]. Additionally, CART cells with the ICOS and 4-1BB intracellular signaling domains showed enhanced efficacy in solid tumors when compared to the 4-1BB-based CART cells [24].

Beside the T cell subtypes, the differentiation status of CART cells also plays a crucial role for therapeutic success. Isolated and ex vivo expanded T cells provide intrinsic properties that have to be considered in cellular immunotherapy. T cells vary in effector function, phenotypic characteristics, and their appearance in peripheral blood (PB) of healthy donors and patients depending on age, previous antigen exposure, and applied cytotoxic therapies due to their differentiation status [25]. In ACT, terminally differentiated CD45RA+ CCR7− T effector-like cells (T_Eff_ cells) demonstrated enhanced in vitro anti-tumor activity, whereas in vivo T cell activation, proliferative capacity, and persistence were impaired [14]. These findings changed the approach and criteria for the selection of specific T cell subsets for ACT and set the focus on less differentiated T cells: naïve-like T cells (T_N_ cells) defined as CD45RA+ CD45RO− CD95− T cells express the lymph node homing markers CCR7 and CD62L, as well as CD28 and CD27 [17]. In contrast, the CD45RA− CD45RO+ CD95+ memory T cells can be divided in CD62L+ CCR7+ T central memory-like cells (T_CM_ cells) and in CD62L− CCR7− T effector memory-like cells (T_EM_ cells) [17]. Stem cell memory-like T cells (T_SCM_ cells) represent a recently described T cell subpopulation resembling T_N_ cells in that they are CD45RA+ CD45RO− CCR7+ and they express memory associated markers, such as CD95, and thereby exhibit properties of stem cells, including high proliferative and self-renewal capacity [25,26]. T_N_ cells and T_SCM_ cells have the capacity to persist and proliferate long-term in vivo after administration to the patient and they can possibly lead to improved clinical outcome [16,26,27]. In particular, the ability of self-renewal and the capacity to differentiate in all memory and effector subpopulations enable T_SCM_ cells to sustain a long-lasting anti-tumor activity by supplying the immune attack with more differentiated T_EM_ cells and T_Eff_ cells and refresh the pool of T cells with new less differentiated T_SCM_ cells and T_CM_ cells [17]. Consequently, transfusion of a high number of less differentiated CART cells is favorable for therapeutic success. The potential of individual T cell subsets is well described in the literature [17]. However, descriptions regarding how the formation of a more favorable cellular composition and T cell phenotype in the final CART cell product can be achieved during the production process are sparse.

## 3. Expression of Exhaustion and Homing Markers on CART Cells

Inhibitory tumor microenvironment binding inhibitory receptors, such as PD-1, CTLA-4, LAG-3, and TIM-3 on T cells might also cause insufficient response rates of CART cells in certain tumor entities, and therefore impair the immune attack [28,29]. A high expression of fatigue-related inhibitory receptors PD-1 and TIM-3 on CD8+ T cells is associated with impairment of the T cell function [30]. The dysfunction of tumor-specific T cells is a dynamic process that leads to antigen-driven differentiation and it is initiated in an early stage of tumorigenesis [31]. Transcriptomic profiling demonstrated that the expression of memory-related genes was enriched in CART cells from CLL patients achieving complete remissions. In contrast, the analysis of CART cells from non-responders revealed an upregulation of genes that mediate T cell differentiation, glycolysis, exhaustion, and apoptosis [32]. Furthermore, a population of less differentiated CD8+ CART cells without PD-1 expression was identified to play a crucial role in tumor control [32]. In addition, lower expression of PD-L1, PD-1, LAG-3, and TIM-3 was observed in lymphoma patients responding to CD19-specific CART cells treatment. Whereas non-responders were expressing higher levels of immune-checkpoint ligands on tumor cells and receptors on immune cells [33]. CART cells can provoke a reversible antigen loss through trogocytosis by transferring the target antigen to T cells, leading to a decrease of target density on cancer cells [34]. Additionally, T cell activity is reduced through the promotion of exhaustion and fratricide T cell killing [34]. It was reported that CART cells encoding a single immunoreceptor tyrosine-based activation motif (ITAM) showed an improved persistence of highly functional CART cells [35]. Strategies that led to a disruption of the interaction between inhibitory T cell receptors and their ligands expressed on cancer cells may improve the therapeutic efficacy of cell-based therapies. The administration of a PD-1 blocking antibody increased the therapeutic efficacy of CART cells [36]. Additionally, it was reported that anti-PD-1 single chain variable fragment (scFv)-producing CART cells mediated potent therapeutic effects when compared to conventional CART cells in preclinical models [37]. While these strategies aim to optimize CART cell therapy in vivo after the administration to the patient, additional strategies are essential that improve the exhaustion status and, in particular, possibly reduce the expression of inhibitory receptors on CART cells during the manufacturing process.

Another challenge is the improvement of CART cell infiltration into the tumor site. The T cell homing is the consequence of multiple molecular interactions. The repression of the anti-tumor immune response of CART cells in the tumor site can be mediated by an immunologic barrier [38]. Different homing properties constitute another distinctive feature of the different T cell subsets. While T_N_ cells, T_SCM_ cells, and T_CM_ cells tend to migrate into lymphoid tissue, the T_EM_ cells and T_Eff_ cells prefer peripheral tissue [25]. A stronger expression of the lymphoid homing marker CD62L and CCR7 on less differentiated T cells is associated with increased anti-tumor activity in preclinical models of ACT and might be beneficial for CART cells [27]. T cell extravasation, homing, and persistence in the tumor microenvironment are essential aspects in overcoming current limitations of CART cell therapy in solid tumors. It was demonstrated that CD28 costimulation could reduce the inhibition of T cell proliferation mediated by the transforming growth factor β (TGFβ) [39]. The overexpression of CXCR2 can improve T cell migration into tumor sites [40]. The overexpression of CCR2b on mesothelin-specific CART cells [41] and GD2-specific CART cells [42] led to enhanced T cell tumor infiltration. It was reported that CD30-specific CART cells expressing CCR4 could mediate an enhanced tumor control in a xenograft model [43]. NKG2D-specific CART cells could recruit and activate endogenous antigen-specific cytotoxic CD8+ cells and CD4+ Th cells in the tumor site in a CXCR3-dependent manner, leading to improved tumor eradication [44]. The modulation and role of specific homing marker expression on CART cells has to be further examined in the future.

## 4. Optimization of the CART Cell Manufacturing Process

**Figure 1:**
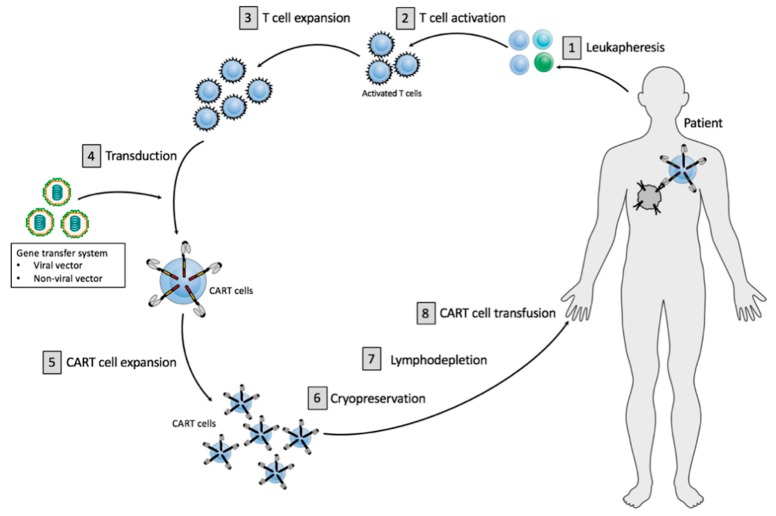
Principles of current CART cell therapy. CART cell production includes initial T cell isolation and enrichment, followed by T cell activation, T cell expansion, gene transfer of a CAR vector and CART cell expansion. The final product is subjected to end-of-process formulation and cryopreservation. Patients usually receive a lymphodepletion before CART cell administration.

The major aspects of the CART cell manufacturing process are relatively standardized, whereas clear differences can be identified in every single manufacturing step (Figure 1). 

The CART cell production process comprises the initial isolation and enrichment of the T cells [1], CART cell preparation, including T cell activation [2], T cell expansion [3], gene transfer of a CAR vector while using viral or non-viral vector systems [4], followed by ex vivo CART cell expansion [5] (Figure 1). The final cell product is subjected to end-of-process formulation and cryopreservation [6] (Figure 1). Quality control testing is performed during the production as well as for the final cryopreserved CART cell product for the integrity of the product. Cancer patients usually receive a lymphodepleting treatment [7] before administration of the finally approved CART cell product [8] (Figure 1).

### 4.1. Isolation and Enrichment of T Vells

Peripheral blood mononuclear cells (PBMCs) are commonly obtained from PB. A ficoll density gradient centrifugation is used for the removal of granulocytes, red blood cells, and platelets [45]. Alternatively, automated cell-washers can be employed to isolate T cells [46]. Further instruments have been developed to facilitate or combine the manufacturing steps in one system, for example, the *Sefia™ Cell Processing System* for isolation, harvesting, and final formulation of cellular products or the *CliniMACS Prodigy^®^* for automated GMP-compliant manufacturing of various cell types [47,48].

The cellular composition at the beginning of the production process can influence the phenotype of the CART cells, as patients with high number of tumor cells in the PB, such as untreated CLL patients, showed low numbers of less differentiated T cells within their PBMCs [49]. Endogenous cellular elements can be a sink for supplemented cytokines and, therefore, may reduce the cytokine-mediated effects on CART cells [50]. Therefore, the selection of CD3+ T cells might be necessary in patients with a high number of circulating tumor cells in the PB. Magnetic bead-based systems, such as the *CliniMACS^®^ system*, with, for example, anti-CD3+, anti-CD4+, or anti-CD8+ microbeads can be used for the selection or depletion of specific T cell types within the PBMCs enabling T cell expansion and administration of the final cell product with a defined CD4:CD8 ratio [45]. A focus was on the development of clinical-scale selection, transduction, and cell expansion of these less differentiated T cells in order to enrich T_N_ cells, T_SCM_ cells, and T_CM_ cells [51,52,53]. The CART cell production from defined T cell subsets seems to be very beneficial. However, a prior selection process can complicate the manufacturing process and the optimal cellular composition at the beginning of CART cell production is not yet defined.

### 4.2. T Cell Activation

T cell activation represents an indispensable step of the CART cell production. Optimal activation should lead to sufficient T cell expansion without causing an immense T cell differentiation or activation induced cell death (AICD). Antigen presenting cells (APCs), such as dendritic cells (DCs), mediates physiological T cell activation. DCs come along with difficult laboratory and clinical application so that DCs are not practical for CART cell therapy [45]. Simplified activation strategies have been developed in order to avoid the usage of APCs as endogenous activators for ex vivo T cell activation.

#### 4.2.1. Anti-CD3/Anti-CD28 Antibodies

An established concept to activate T cells represents the use of unconjugated monoclonal antibodies. Coating of culture dishes or bags can use anti-CD3 monoclonal antibodies (OKT-3) with or without anti-CD28 monoclonal antibodies. More common is the use of anti-CD3/anti-CD28 antibody coated magnetic beads as artificial antigen presenting particles. A strong proliferative signal is provided by the anti-CD3 antibodies, whereas the anti-CD28 antibodies can deliver a potent costimulatory signal [54]. The beads allow for a continuous stimulation of the cells and they can be removed by a strong electromagnet. It was reported that the cytokine production and, thereby, the T cell activation was higher with beads when compared to activation with OKT-3 and interleukin (IL)-2 [52,54]. Furthermore, anti-CD3/anti-CD28 antibody coated magnetic beads may induce the generation of less differentiated and potentially less senescent T cells as well as CART cells with enhanced proliferative capacity and early in vivo anti-tumor responses as compared to stimulation with soluble OKT-3 and high-dose IL-2 [55]. Additional advantages concern the CART cell manufacturing process itself. Enrichment and washing are more simplified, as the beads bound to cells can be magnetically retained. Moreover, the beads without the removal of the beads can perform selection and activation until the end of expansion and the loss of expensive stimulating antibodies during media exchange can be reduced [45]. Therefore, the anti-CD3/anti-CD28 antibody coated magnetic beads are assumed to be the more promising activating strategy. In recent clinical trials, anti-CD3/anti-CD28 antibody coated magnetic beads are frequently used, for example, for the production of *Kymriah^®^* (*Tisagenlecleucel; CTL019*) [56,57] and *Lisocabtagene maraleucel* (*liso-cel; JCAR017*) [20], whereas for the production of *Yescarta^®^* (*Axicabtagene Ciloleucel; KTE-019*) anti-CD3 antibodies with IL-2 are used [58].

It was reported that a specialized polymeric nanomatrix product (*T Cell TransAct*™) conjugated to humanized recombinant CD3 and CD28 agonists together with a serum-free medium (*TexMACS*™) can be used for T cell activation during CART cell production. This strategy led to an increase of T_CM_ cells with high CCR7 and CD62L expression, enhanced IL-2 production, and lower levels of exhausted CD57+ cells when compared to activation with plate-bound anti-CD3/anti-CD28 antibodies [59]. However, PD-1 expression was not significantly influenced. This strategy had no significant influence on the gene transfer efficiency. It was reported that this activation strategy mediated lower expansion, supported the expansion of CD4+ T cells with a CD4:CD8 ratio of average 2:1, and additionally resulted in lower cytolytic activity when compared to activation with the plate-bound anti-CD3/anti-CD28 antibodies [59].

#### 4.2.2. Retronectin

Another activation strategy involves the recombinant human fibronectin fragment RetroNectin^®^ (Retronectin), which is mainly known to mediate increased gene transfer efficiency in retroviral transduction. Its use as a T cell activator is less known. Retronectin together with plate-bound anti-CD3 or anti-CD3/anti-CD28 monoclonal antibodies used for T cell activation during the production of GD2-specific CART cells [60] and CD19-specific CART cells [61] can promote a T_N_ and T_SCM_ cell phenotype. A similar effect was reported for retronectin-mediated T cell activation, together with anti-CD3 antibody or anti-CD3/anti-CD28 antibody coated beads for engineered AcGFP-expressing T cells [62]. Retronectin-based T cell activation can increase the amount of cytotoxic CD8+ T cells and possibly shift the CD4:CD8 ratio towards 1:1, whereas activation with anti-CD3/anti-CD28 induces CD4+ Th cell expansion [60,61,62]. Major disadvantages of retronectin-mediated T cell activation are poorer T cell activation, insufficient T cell expansion, reduced gene transfer efficacy, and reduced cytokine secretion, as it was reported for GD2-specific CART cells [60] and CD19-specific CART cells [61]. Additionally, T cell activation with retronectin has to be performed with caution in patients with a high tumor burden in the PB, as it can activate and stimulate persistent malignant B cells within the cell product, particularly if no T cell selection process was performed prior to T cell activation at the beginning of the production [61].

#### 4.2.3. Artificial Antigen Presenting Cells

In recent studies, the T cell activation for CART cell production have been performed with non-viable artificial APCs presenting a tumor-associated antigen (TAA) to activate T cells in a CAR-dependent manner [63]. For this purpose, K-562 cells were genetically modified in order to co-express costimulatory molecules and a TAA [63]. Lethally irradiated, these modified K-562 cells can be used for numerical expansion of CART cells. The advantages of this strategy are that no expression of HLA-A or HLA-B molecules is described, and that good manufacturing practice (GMP)-compliant cultivation can be performed [63]. Additionally, these artificial APCs only stimulate the TAA-specific CART cells.

In summary, the applied activation strategy can positively influence the cellular composition and phenotype of the cell product. The predominant T cell activation strategy in recent studies is the use of anti-CD3/anti-CD28 antibody coated magnetic beads, followed by monoclonal antibodies. However, new alternative strategies are in development. The optimal strategy depends on tumor entity and tumor burden in the PB.

### 4.3. Gene Transfer System

Non-viral or viral gene transfer vectors transferring the corresponding genetic information into the T cells mediate CAR expression on the T cell surface. Plasmid-based transposon/transposase systems and viral vectors, including gamma-retroviral and lentiviral vectors as well as genome editing and electroporation of naked DNA are applied for gene delivery in CART cell therapy.

#### 4.3.1. Viral Transduction

Virus-based gene delivery systems are commonly used and they can achieve high transduction efficiency rates [45]. Among the most frequently used viral vector systems are gamma-retroviral vectors and lentiviral vectors, which both belong to the family of retroviruses [64]. Retroviruses mediate a stable long-term gene expression, as the produced viral DNA is integrated into the host DNA [54]. Lentiviruses need regulatory genes to neutralize the host cell defense and weaken the immune response, as well as to regulate viral replication [64]. The risk of insertional mutagenesis and oncogenicity seems to be low with lentiviral vectors [64]. Almost no genotoxic effects from gene transfer into differentiated cells, including T cells, are known. Only a few cases of virus-mediated transformation in patients treated with genetically modified T cells have been reported so far [65,66,67]. A lentiviral vector-mediated insertion of the CAR transgene was observed in a CLL patient treated with CD19-specific CART cells, leading to a disruption of the methylcytosine dioxygenase TET2 gene [65]. These TET2-disrupted CART cells showed a modified T cell differentiation leading to a central memory phenotype at the maximum of proliferation [65]. Although insertional mutagenesis is undesirable, the described TET2 modification could be used for an optimization of CART cell therapy. An additional case of clonal expansion was seen in a patient that was treated with CD22-specific CART cells caused by lentiviral vector-mediated integration in the CBL gene that is important for the regulation of T cell responses [66]. Furthermore, insertional mutagenesis led to tumor escape in a patient relapsing after treatment with CD19-specific CART cells with a CD19-negative leukemia [67]. In this case, the CAR gene was unintentionally introduced into a single leukemic B cell during the CART cell production process, which causes a disguise from recognition [67]. To our knowledge, no accidental insertional has been yet reported for gamma-retroviral vectors used for CART cell therapy. Therefore, gamma-retroviruses are still widely used and seen as a safe vector system for clinical ACT.

Whereas, for the production of retroviral vectors stable packaging cell lines can be used, the production of lentiviral vectors requires large amounts of plasmid DNA for transient transfection [68]. A prerequisite for efficient viral gene delivery is the presence of dividing T cells after T cell activation particularly for retroviral gene transfer [69]. The intensive and expensive vector production is a major disadvantage of viral gene transfer systems, as appropriate clean room facilities and the performance of vector release testing for the retrovirally or lentivirally transduced cells are required [54]. This has become a major bottle neck, even for big pharma in this field. 

Lentiviral transduction is the predominantly used viral gene delivery system and, for example, used for the production of *Kymriah^®^* (*Tisagenlecleucel*) for the treatment of ALL [4] or DLBCL [5] and *Lisocabtagene maraleucel* (*liso-cel; JCAR017*) for the treatment of r/r aggressive NHL [20]. Retroviral transduction was performed, for example, in the ZUMA-1 trial with *Axicabtagene Ciloleucel* for the treatment of r/r large B cell lymphoma [58,70]. Viral vectors both mediate sufficient gene transfer efficiency and lead to safe products. However, viral vector production remains to be very labor- and, therefore, cost-intensive aspect in CART cell production.

#### 4.3.2. Plasmid-Based Gene Delivery

Transposons/transposase systems constitute an alternative strategy for non-viral CAR gene delivery. The “Sleeping Beauty” transposon/transposase system was employed for CART cell manufacturing [71]. This system consists of two DNA plasmids, one containing the transposon encoding the CAR transgene and a second expressing the transposase that is necessary for excision and insertion of the transgene [69,72]. The use of a transposon system can increase the gene transfer efficiency when compared to electroporation of naked DNA, revealed promising results for CART cell therapy and it represents an economically beneficial strategy [45]. The advantage of this plasmid-based gene delivery for CART cell therapy is a less expensive and labor-intensive production, as no GMP-grade virus generation is necessary [69].

Plasmid electroporation was mainly used with 1st generation (1G) [73] and 3rd generation (3G) CART cells [74]. The first clinical usage of the “Sleeping Beauty” transposon/transposase system for CART cell therapy yielded encouraging results [75].

Analyses of the applied gene transfer system currently concentrate on transduction and clinical efficacy, safety, and costs. The optimal gene transfer system is not yet defined, and further investigation is necessary.

#### 4.3.3. Genome Editing

Genome engineering tools, in particular, CRISPR/Cas9-based gene editing, represent an evolving field for CAR-based therapies, enabling an efficient sequence-specific intervention in human cells [76]. The CRISPR/Cas9 technology enables the specific genomic disruption of multiple gene loci. The CRISPR/Cas9 system combined with an adeno-associated virus (AAV) vector repair matrix was applied for the integration of the CAR encoding DNA into the T cell receptor α constant (TRAC) locus provoking an uniform expression of the CAR, an improvement of T cell potency, and an inhibition of T cell differentiation as well as of exhaustion [77]. Additionally, it was reported that CRISPR/Cas9-mediated genome editing and lentiviral transduction was applied to produce PD-1 deficient CD19-specific CART cells, leading to enhanced anti-tumor and therapeutic efficacy [78]. Although multiple challenges, including efficiency, safety, and scalability, are a matter of concern, CRISPR/Cas9-enhanced immune-gene cell therapy might further improve CART cell therapies [76]. Nevertheless, the full potential of genome editing in the context of CART cell-based immunotherapy is not fully utilized and it has to be further examined in human clinical studies.

### 4.4. CART Cell Construct

**Figure 2:**
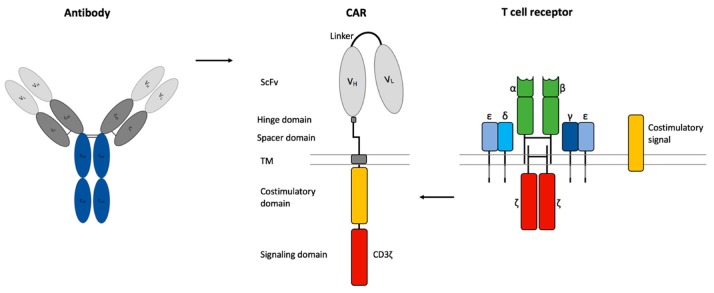
Chimeric antigen receptor (CAR) design. CARs consist of a single chain variable fragment (scFv) of an antibody, a non-signaling extracellular spacer and hinge domain, a transmembrane (TM) domain, an intracellular CD3ζ signaling domain from the T cell receptor and a costimulatory domain.

The optimal composition of the CAR is crucial for efficient CART cell-based cancer immunotherapy. CARs contain a scFv of an antibody as an extracellular binding domain for HLA-independent antigen recognition, a transmembrane (TM) domain, and a CD3ζ chain as an intracellular signaling domain [79] (Figure 2). Additional stability of the CAR can be obtained by a non-signaling extracellular spacer domain between the scFv and the TM domain [80]. The length and composition of the spacer domain can influence the CART cell function independently of the intracellular domain [80,81]. The spacer domain often consists of an IgG hinge domain and a CH2-CH3 domain of an IgG-Fc [79].

**Figure 3:**
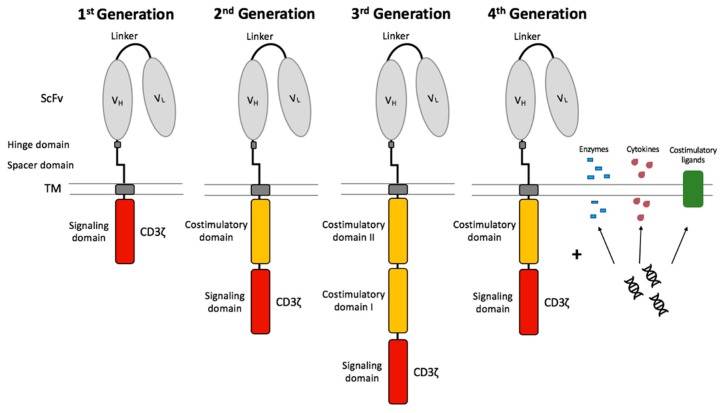
Chimeric antigen receptor generations. The 1st generation CART cells induced T cell activation only by the primary signal via the CD3ζ signaling domain. CART cells were further developed by integration of a costimulatory domain in 2nd generation CART cells. The 3rd generation CART cells consist of two costimulatory domains. The future 4th generation CART cells combine the vector with enzymes, cytokines, and costimulatory ligands.

The CAR design has been further developed over several generations since its introduction (Figure 3). 1G CARs were designed without a costimulatory domain and induced T cell activation only by the primary signal via the CD3ζ signaling domain. CART cells relying only on CD3ζ for signaling showed low cytokine production capacities, insufficient T cell expansion, and quickly became anergic [82,83]. These CD3ζ-based CART cells dispose of a sufficient antigen-specific cytotoxic capacity, however the T cell expansion was weak [79]. Therefore, the clinical results of patients suffering from ovarian cancer [84], NHL [85], and neuroblastoma [86] treated with CD3ζ-based CART cells were also limited. 2nd generation (2G) CARs were developed in order to achieve long-term persistence and expansion as well as to prevent AICD and anergy [87]. The far-reaching change consisted in the integration of a costimulatory domain, such as CD27 [88], CD28 [89,90], CD134 (OX40) [91], or CD137 (4-1BB) [92,93]. This modification improved the in vivo properties of CART cells and protected CART cells from AICD [94]. In vivo persistence was significantly influenced by the inserted costimulatory domains [95]. It has been described that CD28, as a costimulatory domain, supports stronger T cell expansion and improved tumor eradication [89,90], whereas 4-1BB, as a costimulatory domain, is associated with prolonged persistence and ameliorates the development of exhaustion [93]. It was demonstrated that tonic CAR CD3ζ phosphorylation can provoke early exhaustion of CART cells that limits antitumor efficacy [93]. Additionally, it was shown that CD28 costimulation increases and 4-1BB costimulation decreases exhaustion induced by ongoing CAR signaling [93]. While 2G CAR only contain one costimulatory domain (CD28 or 4-1BB), the 3G CARs contain a second costimulatory signal [96,97]. The combination of two costimulatory domains in a 3G CAR might have the potential to combine these two advantages. The simultaneous infusion of 2G (CD28) and 3G (CD28/4-1BB) CD19-specific CART cells in patients showed that the 3G CART cells had superior expansion and persistence properties [98]. Furthermore, it was shown that the intracellular signaling activity of 3G CART cells was higher than that of 2G CART cells, and probably led to superior cell proliferation [96]. First, clinical studies with anti-CD19 3G CART cells demonstrated efficacy and safety in patients with B cell malignancies [99].

Further development of the CAR led to 4th generation (4G) CARs and T cells redirected for universal cytokine-mediated killing (TRUCKs). These novel 4G CARs express molecules due to supplementary genetic modifications within the CAR construct to improve the therapeutic efficacy of the CART cell therapy [100]. TRUCKs are CAR-redirected vehicles that can produce and release a transgenic product, for example, a pro-inflammatory cytokine at the tumor site [100]. The recruitment and activation of other components of the immune system can be achieved through the additional expression of costimulatory ligands, such as 4-1BB-L [101] and CD40-L [102], or proinflammatory cytokines, such as IL-15, IL-7, and IL-21 [103] leading to superior anti-tumor cytotoxicity. It was reported that CD19-specific CART cells secreting IL-12 eradicate established tumor disease without a prior conditioning regime [104]. It has to be mentioned that tumor-targeted IL-12 secreting T cells became resistant against T_reg_ cell-mediated inhibition [104]. The integration of a CAR-inducible IL-12 cytokine cassette leads to the secretion of IL-12 after CAR signaling, and thus to the accumulation and maintenance of therapeutic levels of the cytokine in the targeted tissue, leading to the destruction of TAA-expressing cells and TAA-negative tumor cells [105,106]. A disadvantage is that only the antigen-expressing tumor sites can initiate the release of IL-12. This strategy has to be applied with caution: use of cytokines with safe toxicity profiles and controlled release of cytokines are needed [107]. Moreover, it was reported that armored CART cells that have been modified to express degrading enzymes showed enhanced capacity to infiltrate tumor sites [108].

Extensive research is currently ongoing for further optimizing CAR constructs. Most of the protocols for CART cell generation are optimized for 2G and 3G CARs. Further analysis will be necessary if novel production protocols have to be developed for these novel CART cell therapy approaches.

### 4.5. T Cell Expansion

During the expansion of CART cells, the number of cells continuously increases, so that the volume of culture medium has to be modified by means of more or larger tissue culture flasks or plates. This immensely complicates the manufacturing process and it is not compatible with large-scale production. Therefore, static culture bags have been developed, allowing a less manual open-handling, as the connection by tubes can be performed under sterile conditions [45]. An alternative method represents a rocking motion bioreactor, such as the *Xuri™ Cell Expansion System* and *WAVE*^TM^ *Bioreactor System*, which utilize a perfusion regime to add nutrients as well as remove growth-inhibiting substances, thereby simplifying the manufacturing process [109,110].

#### 4.5.1. Stimulation with Cytokines

Besides the CAR vector and the T cell activation strategy, ex vivo stimulation with supplemented γ-chain cytokines during the CART cell production process is another important factor that influences the composition, quality, and phenotype of the final CART cell product. The receptors for cytokines of the γ-chain family, such as IL-2, IL-4, IL-7, IL-9, IL-15, and IL-21, have a common CD132 or γ-chain. The two most commonly used strategies for CART cell production are based on either IL-2 or IL-7, with or without IL-15. So far, IL-2 was primarily used for T cell expansion in previous clinical studies [3,45,46]. For example, IL-2 is supplemented for the production of *Yescarta^®^* (*Axicabtagene Ciloleucel*) [70]. However, ex vivo T cell expansion in the presence of IL-2 can lead to a more differentiated and exhausted phenotype and it can reduce T cell persistence [111]. Expansion with IL-7/IL-15 was shown to enhance activation and proliferation when compared to IL-2 [60]. Additionally, it was reported that a combination of IL-7/IL-15 promotes the survival and maintenance of less differentiated T cells, such as T_N_ cells and T_SCM_ cells with high CD62L and CCR7 expression [112,113,114]. Moreover, the supplementation of IL-7-/IL-15 mediated a higher expansion of CD4+ and CXCR3+ CD19-specific CART cells [114] and NY-ESO-1-specific T cells [115] when compared to expansion with IL-2. It was reported that supplementation of IL-15 alone can lead to reduced exhaustion marker expression, an increase of anti-apoptotic properties, improved proliferation, and preservation of a T_SCM_ phenotype as compared to IL-2 [116]. Moreover, IL-15 induced a reduction in mTORC1 activity, a decrease of glycolytic enzyme expression, and enhanced mitochondrial fitness, thus leading to prevention of T cell differentiation [116].

IL-21 is another important member of the γ-chain family. It was reported that TILs expanded with IL-21 showed a CD27+ CD28+ less differentiated phenotype with enhanced cytotoxic capacity [117]. In adoptive cell transfer, IL-21 can suppress antigen-induced differentiation of CD8+ T cells, whereas IL-2 and IL-15 enhance the differentiation into terminally differentiated T_Eff_ cells [118]. Furthermore, IL-21 mediated a higher expression of CD62L when compared to IL-2 and IL-15 as well as enhanced antitumor activity [118]. The adoptive transfer of IL-21-stimulated CD19-specific CART cells resulted in enhanced control of B cell malignancies in preclinical models [119].

In summary, the supplementation of cytokines during ex vivo expansion of CART cells is essential and indispensable for CART cell manufacturing protocols. Current studies mainly rely on IL-2, IL-7, IL-15, and IL-21. The optimal cytokine composition, as well as the role of other cytokines for CART cell generation, is not clearly defined yet.

#### 4.5.2. Inhibition of Specific Signaling Pathways

**Figure 4:**
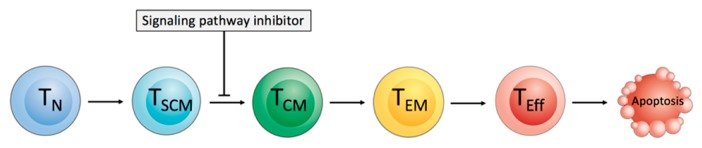
Inhibition of signaling pathways for interrupting of the T cell differentiation process. The differentiation of naïve-like T (T_N_) cells and stem cell memory-like T (T_SCM_) cells into T central memory-like (T_CM_) cells, T effector memory-like (T_EM_) cells, and highly differentiated T effector-like (T_Eff_) cells can be interrupted by molecules inhibiting key metabolic and developmental pathways.

The supplementation of pathway inhibitors during ex vivo T cell expansion might cause an interruption of T cell differentiation by the inhibition of specific signaling pathways, and thus shift the T cell phenotype in the final CART cell product towards a less differentiated phenotype [120]. The possible targets include GSK3β, mTOR, AKT, and PI3K for specific pathway inhibition (Figure 4).

It has been reported that the induction of the WNT-β catenin signaling pathway by GSK3β inhibition might interrupt the T cell differentiation process and can generate CD8+ T_SCM_ cells [121].

The PI3K-AKT-mTOR signaling pathway is crucial for T cell activation, survival, expansion, migration, function, and differentiation [122]. mTOR plays a central role in T cell memory formation, and the mTOR inhibitor rapamycin can mediate, in preclinical evaluation, a higher number of T memory cells, increased expression of the lymph node homing marker CD62L, and the anti-apoptotic molecule Bcl-2 [123]. It was reported that the supplementation of IL-15 during ex vivo CART cell expansion could reduce mTORC1 activity and preserve a less differentiated phenotype [116]. The IL-15-mediated preservation of a less differentiated T cell phenotype is most likely caused by reduced mTORC1 activity, as CART cells ex vivo expanded with IL-2 and the mTORC1 inhibitor rapamycin showed similar phenotypic features like CART cells expanded with only IL-15 [116]. It was shown that adoptively transferred T cells showed improved anti-tumor activity after ex vivo AKT inhibition [124,125].

The B cell receptor (BCR) pathway inhibitor idelalisib, which is an inhibitor of phosphatidylinosit-3-kinase p110δ (PI3Kδ), is currently approved for the treatment of patients with CLL and follicular lymphoma. In addition to eliminating malignant B cells, idelalisib can degrade T_reg_ cells, and thereby reverse the immune tolerance of cancer cells [126,127]. The ex vivo treatment of CART cells with a PI3Kδ inhibitor mediated higher amounts of less differentiated CCR7+ CD62L+ T cells and improved the functional capacity of mesothelin-specific [128], CD33-specific [129], and CD19-specific CART cells [49]. In healthy donors, the supplementation of IL-7/IL-15 during the production process led to a very balanced CD4:CD8 ratio. However, CLL patient samples without prior T cell selection showed an unbalanced CD4:CD8 ratio with predominant CD4+ T cells that could be approximated towards a 1:1 ratio by the supplementation of idelalisib during the manufacturing process [49]. The reduced expression of exhaustion markers was another positive effect of the application of a PIK3δ inhibitor during CART cell production [49,128]. The use of antagonists against PI3Kδ and the vasoactive intestinal peptide (VIP) during ex vivo expansion of DLBCL patient T cells led to the inhibition of the terminal T cell differentiation, reduced PD-1 expression, and improved T cell persistence in immune-deficient mice [130]. The addition of these antagonists improved the expansion and gene transfer efficacy of human anti-CD5 CART cells, as well as their cytotoxic capacity against CD5+ lymphoma cells [130]. This showed that a synergistic blockade is also a promising strategy for improving the expansion and functional capacity of ex vivo expanded antigen-specific T cells [130]. Furthermore, it was reported that the B cell adaptor for PI3K (BCAP) is also a regulator of CD8+ T cell differentiation and might be another target for inducing the formation of specific T cell subpopulation [131].

The inhibition of the PI3K/AKT/mTOR pathway can lead to the down-regulation of c-Myc. The treatment of T cells with the bromodomain and extra-terminal motif (BET) bromodomain inhibitor downregulating c-Myc-dependent target genes [132] resulted in the enhanced expansion of CD8+ T_SCM_ cells and T_CM_ cells, improved the persistence and anti-tumor activity of CART cells in an ALL model [133]. An increase of T_N_ cells and T_CM_ cells has also been reported in CD33-specific CART cells that were treated five days after T cell activation for four days with BET inhibitors JQ-1 or iBET [134].

In summary, the ex vivo treatment with specific pathway inhibitors during the CART cell manufacturing process might have a positive effect on CART cells and can, therefore, improve the final CART cell product. The optimization of the usage of signaling pathway inhibitors for clinical application will be the next step in further enhancing the efficacy of CART cell therapy.

### 4.6. Cryopreservation

The cryopreservation of CART cells at the end of production is mandatory for quality control tests in most currently applied CART cell therapy approaches and it enables the transportation of the final product from manufacturing sites to clinical centers. Moreover, the administration of the product is more flexible, and patients could possibly receive multiple CART cell treatments. It was reported that the cryopreservation of CART cells for up to 90 days did not hamper the viability, recovery, and gene transfer efficacy of the cryopreserved CART cells [135]. Although the functionality of cryopreserved CART cells directly after thawing was reduced, an overnight incubation at 37 °C led to recovery from the harsh freeze-thaw process with restored functionality of the CART cells [135]. Additionally, CART cells that have been cryopreserved and thawed immediately before transfusion showed similar in vivo persistence and efficacy as fresh CART cells [136]. In summary, cryopreservation is a regularly applied manufacturing step with obviously no significant impairment of the CART cell product.

## 5. Conclusions and Future Perspective

CART cell therapy represents a promising new therapeutic option for patients with hematological malignancies and it might also become a therapeutic option for patients with solid tumors soon. Even though FDA and EMA both approve first CART cell products, the expensive and highly variable manufacturing processes are still a matter of debate. Improved CART cell therapy might be achieved by the transfusion of a CART cell product with a favorable phenotype, including less differentiated CART cells. Furthermore, costs may be reduced through more efficient production protocols. The potential of anticancer immunotherapy improvement by optimizing the ex vivo expansion conditions during the CART cell manufacturing process has not yet been fully exploited. Therefore, further efforts are mandatory for standardizing and optimizing CART cell production protocols.

## Abbreviations

1G	1st generation
2G	2nd generation
3G	3rd generation
4G	4th generation
AAV	Adeno-associated virus
ACT	Adoptive cell therapy
AICD	Activation induced cell death
ALL	Acute lymphoblastic leukemia
APCs	Antigen presenting cells
BCAP	B cell adaptor for phosphoinositide 3-kinase
BCMA	B cell maturation antigen
BCR	B cell receptor
BET	Bromodomain and extra-terminal motif
CAR	Chimeric antigen receptor
CLL	Chronic lymphocytic leukemia
CTLA-4	Cytotoxic T-lymphocyte-associated Protein 4
DCs	Dendritic cells
DLBCL	Diffuse large B cell lymphoma
EMA	European Medicines Agency
FDA	Food and Drug Administration
GMP	Good manufacturing practice
HLA	Human leukocyte antigen
ICOS	Inducible T cell costimulator
IL	Interleukin
ITAM	Immunoreceptor tyrosine-based activation motif
NHL	Non-Hodgkin lymphoma
LAG-3	Lymphocyte-activation gene-3
PB	Peripheral blood
PBMC	Peripheral blood mononuclear cells
PD-1	Programmed cell death protein 1
PI3K	Phosphoinositide 3-kinase
PMBCL	Primary mediastinal B cell lymphoma
r/r	relapsed/refractory
scFv	Single chain variable fragment
TAA	Tumor-associated antigen
TCR	T cell receptor
T_CM_ cell	Central memory-like T cell
T_Eff_ cell	Effector-like T cell
T_EM_ cell	Effector memory-like T cell
TGFβ	Transforming growth factor β
Th cell	T helper cell
TILs	Tumor-infiltrating lymphocytes
TIM-3	T cell immunoglobulin and mucin-domain containing-3
T_N_ cell	Naïve-like T cell
TM	Transmembrane
TRAC	T cell receptor α constant
T_reg_ cell	Regulatory T cell
TRUCK	T cells redirected for universal cytokine killing
T_SCM_ cell	Stem cell memory-like T cell
VIP	Vasoactive intestinal peptide
